# Data on perception of faculty members on the influence of faculty support initiatives on the efficacy of job responsibilities

**DOI:** 10.1016/j.dib.2018.06.065

**Published:** 2018-06-22

**Authors:** H.O. Falola, A.A. Adeniji, A.O. Osibanjo, O.A. Oludayo, O.P. Salau

**Affiliations:** Department of Business Management, Covenant University, Ota, Nigeria

## Abstract

The main objective of this survey is to present data on the perception of the influence of faculty support initiatives on efficacy of job responsibilities using six private university in Nigeria as case study. The population of the study included all faculty members of selected private universities with a total of 1912 faculty members. A sample size of 500 were selected to participate in the survey based on the outcome of the sample size determination formula suggested by Falola et al. (2016) [5]. The validity and reliability of the research instrument were carried out. Regression analysis and structural equation modeling were used as statistical tool of analysis. It is believed that when the data is analysed, it will give insight into how faculty support initiatives of various universities can help in enhancing the effectiveness of job responsibilities.

**Specification Table**

**Table:** 

**Subject area**	Management
**More specific subject area**	Human Resource Management
**Type of data**	Table
**How data was acquired**	Copies of questionnaire were administered to faculty members of some selected private universities in Nigeria
**Data format**	Raw, analyzed and statistical data
**Experimental factors**	Stratified and Simple random sampling of faculty members of some selected universities.
**Experimental features**	The perception of faculty members on the influence of faculty support initiatives on the efficacy of job responsibilities
**Data source location**	South west Nigeria
**Data accessibility**	All the data are included in this article

**Value of the data**

• University management can have insight into which of the faculty support initiatives mostly predicts efficacy of faculty responsibilities.
• If the data is properly analysed, it can provide a platform upon which universities take decisions that will completely restore the dignity of Nigerian Universities. See [3], [5] for similar data.
• The data provided here can be used for decision making purposes.
• This can be used as a platform upon which management of the universities and other stakeholders in the education sector formulate policies.
• The questionnaire can be adopted or adapted for a similar studies.

## 1 Data

The data presented in this study is quantitative in nature. It comprises raw statistical data on the influence of faculty support initiatives on the efficacy of job responsibilities. The study is descriptive in nature. Statistical Package for Social Sciences (SPSS) was used for the coding of the data collected. The population, sample size and response rate of questionnaire administered as well as demographic characteristics of the respondents are depicted in Table 1, Table 2. Meanwhile, Table 3, Table 4 show the descriptive outputs of the measures of independent and dependent variables while Table 5 shows the standardized regression weights of the structural equation modelling outputs as generated by AMOS 22. It is also important to note that 5-point Likert scale of questionnaire was used for the collection of data from the respondents as suggested by [1], [2], [3]. However, the relationship and resultant effect of faculty support initiatives and efficacy of job responsibilities is depicted in Fig. 3. The data can provide a deep insight that will help the management of the universities and other stakeholders in the education sector to formulate policies and decision that will help in repositioning the university education in Nigeria.

**Table 1:** Sample size and questionnaire administration.

**S/N**	**University**	**Population**	**Sample**	**No. of returned questionnaire**
1.	University A	362	95	88
2	University B	377	99	91
3	University C	200	52	46
4	University D	476	124	101
5	University E	296	77	70
6	University F	201	53	47
	**Total**	**1912**	**500**	**443**

**Table 2:** Demographic characteristics of academic staff.

			University						Total
			University A	University B	University C	University D	University E	University F	
**Gender**									
Gender		Male	73	84	37	82	48	40	364
		Female	15	7	9	19	22	7	79
Total			88	91	46	101	70	47	443
**Age**									
Age		18–30	6	7	2	14	5	3	37
		31–40	41	24	5	23	21	11	125
		41–50	22	29	16	25	29	32	153
		51-Above	19	31	23	39	15	1	128
Total			88	91	46	101	70	47	443
**Marital status**									
Marital Status		Single	22	7	2	13	5	3	52
		Married	66	81	44	87	61	44	383
		Others	0	3	0	1	4	0	8
Total			88	91	46	101	70	47	443
**Current rank**									
Rank		Prof	15	17	12	20	5	5	74
		Ass Prof.	4	14	10	13	9	6	56
		Snr. Lecturer	18	23	10	27	17	14	109
		Lecturer I	28	14	5	18	29	13	107
		Others	23	23	9	23	10	9	97
Total			88	91	46	101	70	47	443

**Table 3:** Descriptive statistics of items measuring faculty support initiatives.

**Descriptive statistics**								
	N	Mean		Std. Dev	Skewness		Kurtosis	
	Statistic	Statistic	Std. Error	Statistic	Statistic	Std. Error	Statistic	Std. Error
Conference Support (CS)	443	4.1151	.02631	.55383	− .798	.116	.793	.231
Research Grant (RG)	443	3.3634	.04058	.85417	− .252	.116	− .406	.231
Research Leave with Pay (RL)	443	4.1941	.02242	.47187	.017	.116	.480	.231
Publication Support (PS)	443	3.7675	.02622	.55185	− .021	.116	.192	.231
Valid N (listwise)	443

**Table 4:** Descriptive statistics of items measuring efficacy of faculty job responsibilities.

**Descriptive statistics**								
	N	Mean		Std. Deviation	Skewness		Kurtosis	
	Statistic	Statistic	Std. error	Statistic	Statistic	Std. error	Statistic	Std. Error
Research Outputs (RO)	443	4.2009	.02510	.52826	− .244	.116	− .149	.231
Administrative Role (AR)	443	3.8450	.02933	.61737	− .241	.116	.142	.231
Knowledge Sharing (KS)	443	4.1512	.02199	.46274	− .304	.116	.466	.231
Valid N (listwise)	443

**Table 5:** Standardized regression weights.

			Estimate	S.E.	C.R.	P
AR	<---	PS	0.530	0.087	5.109	***
KS	<---	PS	0.464	0.074	3.472	***
KS	<---	CS	0.503	0.043	5.372	***
RO	<---	RL	0.274	0.065	2.840	.005
AR	<---	RG	0.220	0.044	3.144	.002
RO	<---	CS	0.318	0.035	3.547	***
CS3	<---	CS	0.700	0.026	3.950	***
CS2	<---	CS	0.704	0.061	14.425	***
CS1	<---	CS	0.984	0.045	15.088	***
RG3	<---	RG	0.709	0.029	15.751	***
RG2	<---	RG	0.891	0.086	14.234	***
RG1	<---	RG	0.747	0.071	14.017	***
RL3	<---	RL	0.731	0.056	13.801	***
RL2	<---	RL	0.794	0.121	11.039	***
RL1	<---	RL	0.615	0.088	10.436	***
PS3	<---	PS	0.701	0.055	9.833	***
PS2	<---	PS	0.778	0.105	9.661	***
PS1	<---	PS	0.358	0.087	5.552	***
RO1	<---	RO	0.350	0.069	1.443	***
RO2	<---	RO	0.647	0.319	5.406	***
RO3	<---	RO	0.662	0.330	5.723	***
AR1	<---	AR	0.488	0.341	6.040	***
AR2	<---	AR	0.551	0.155	5.895	***
AR3	<---	AR	0.571	0.165	6.276	***
KS3	<---	KS	0.440	0.175	6.657	***
KS2	<---	KS	0.508	0.178	6.146	***
KS1	<---	KS	0.441	0.139	6.125	***

Table 1 shows the population, sample size and number of returned questionnaire of each university. Meanwhile, it is also important to state here that universities B, D and F are faith based while universities A, C and E are owned by group of individuals.

Table 2 shows the cross-tabulations of the demographic characteristics of the faculty members of the selected universities. If this is interpreted, it will give clear understanding of the composition of the respondents and this can be used for decision making purposes and can as well be leveraged on for further investigation.

**Fig. 1:**
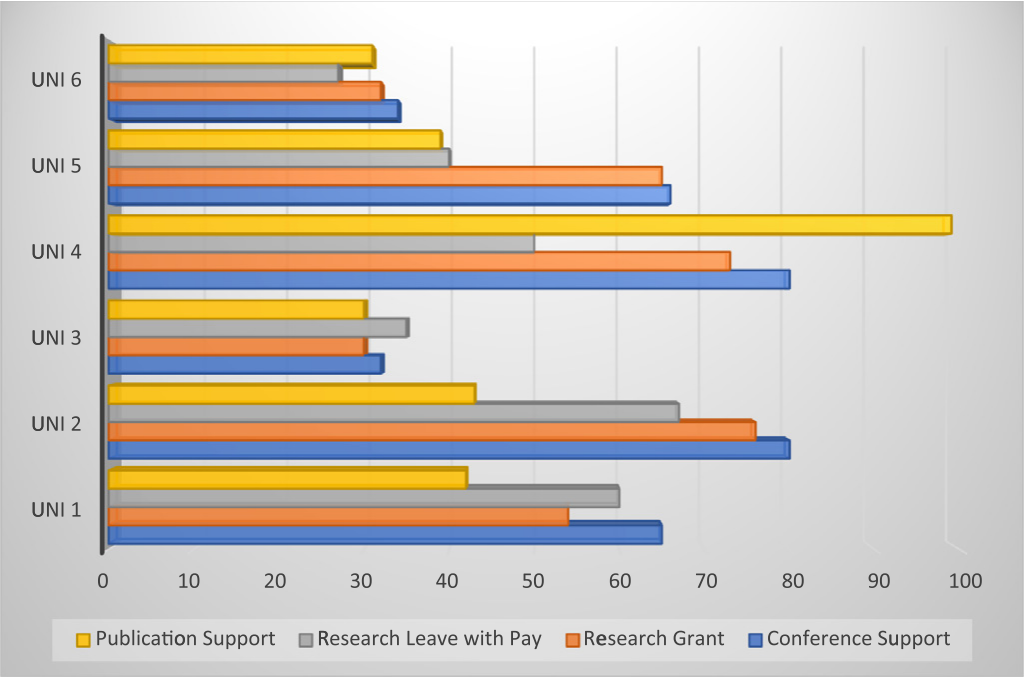
Faculty support initiatives of each university.

Table 3 and Fig. 1 above shows the descriptive statistics of specific items such as conference support, research grant, research leave with pay, publication support that were used to measure university support initiatives for effective job performance in the core areas of faculty responsibilities. Meanwhile, x-axis score of Fig. 1 shows the level to which respondents of each university agreed with the specific construct used for the measurement of faculty support initiatives. The data if analyse and properly interpreted will help to determine which of the support is more effective in driving job performance.

**Fig. 2:**
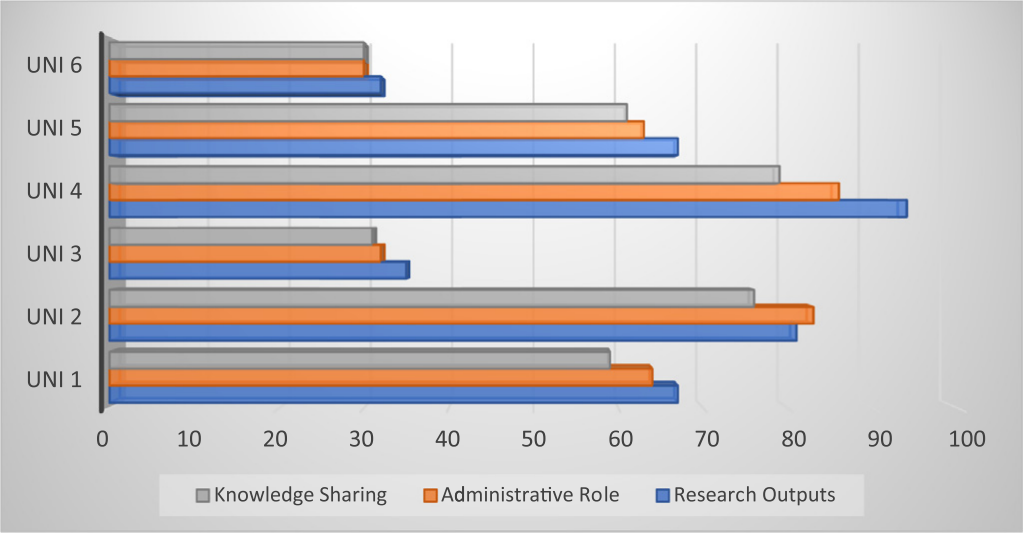
Efficacy of job responsibilities.

**Fig. 3:**
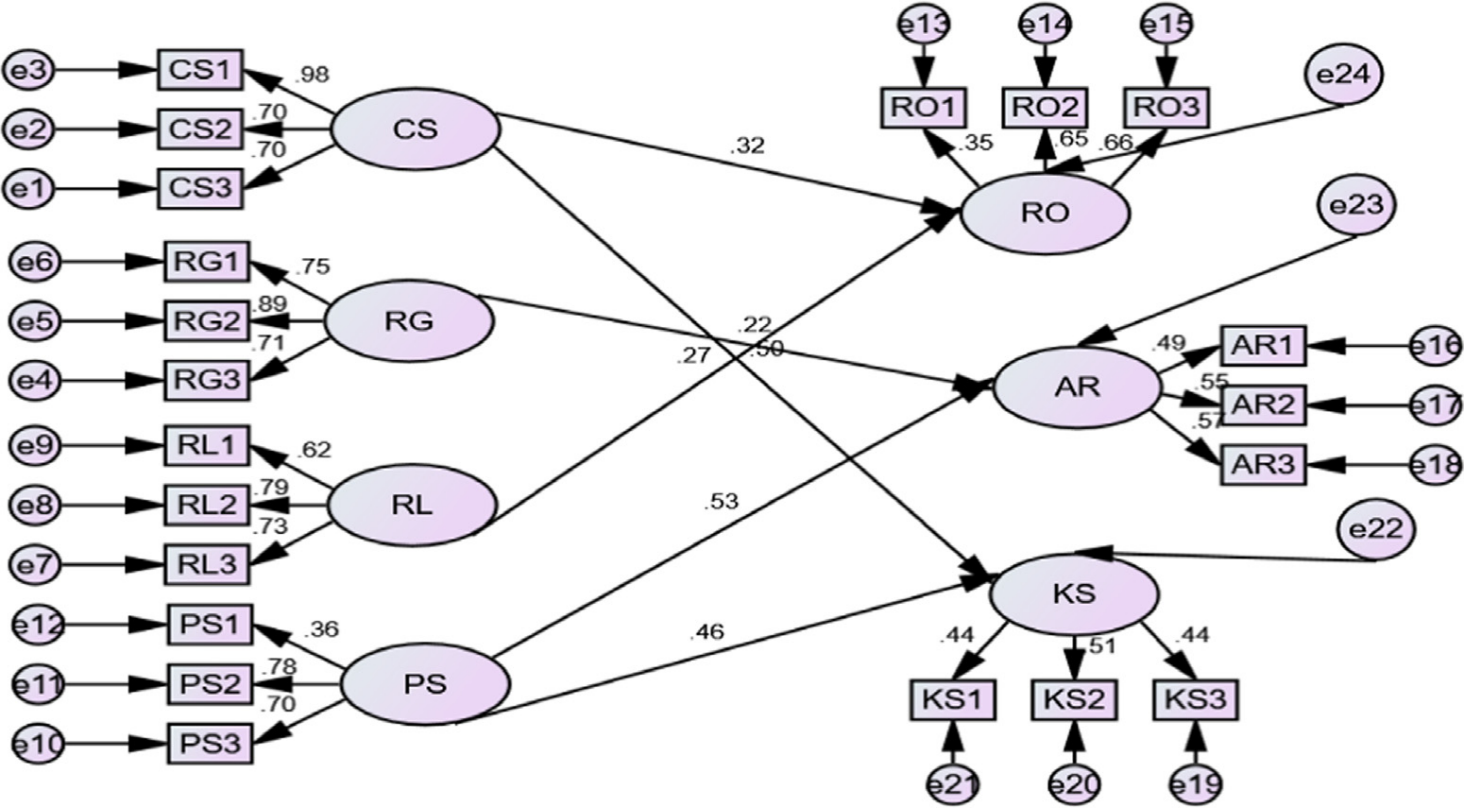
Faculty support initiatives and efficacy of job responsibilities model.

Table 4 and Figs. 2 and 3 show the description statistics of the specific construct used to measure the efficacy of faculty job responsibility such as research outputs, administrative role and knowledge sharing. Meanwhile, x-axis score of Fig. 2 shows the level to which respondents of each university agreed with the specific construct used for the measurement of job responsibilities. If the data presented for both independent and dependent variables are linked together, it will help to determine the level of prediction of each of the constructs.

## 2 Experimental design, materials and methods

Six best private universities as ranked by National Universities Commission, and webometric ranking were selected from Southwest Nigeria. What informs the choice of the best six private universities in southwest Nigeria was because of their outstanding performance when it comes to research and innovation. The researchers wanted to find out the institutional supports given to the Academic Staff that might be responsible for their performance. Data were collected from the sample of four hundred and forty three faculty members across all the colleges with the aid of structured questionnaire designed by the researcher based on the similar studies of [4], [5], [6], [7]. Stratified and simple random sampling techniques were used in order to ensure that every faculty has equal chance of been selected. The data presented information on questions related university support initiatives and efficacy of job performance. Meanwhile, the researchers also sought for the permission of the management of the selected universities before the questionnaire were administered to the faculty members of their institutions. In addition, every faculty member was adequately informed about the objective of the study and they were equally given opportunity to stay anonymous and their responses were treated with upmost confidentiality.
