# Supplementary material for: Data on perception of faculty members on the influence of faculty support initiatives on the efficacy of job responsibilities
Source: Data Brief. 2018 Jun 22;19:1594–9. doi: 10.1016/j.dib.2018.06.065 (PMC6141862; doi:10.1016/j.dib.2018.06.065)
Supplement: Supplementary file 1 — Transparency document [file mmc1.doc]

**Falola Hezekiah Olubusayo (Ph.D)**

Department of Business Management

College of Business and Social Sciences,

Covenant University, Ota, Ogun State, Nigeria

[hezekiah.falola@covenantuniversity.edu.ng](mailto:hezekiah.falola@covenantuniversity.edu.ng)

+234 703 5518 559

**May 17, 2018**

The Editor,

Data in Brief

Dear Sir,

**DECLARATION OF CONFLICT OF INTEREST**

I, Dr. Falola H.O and my colleagues write to declare that there is no conflict of interest traceable to our data paper “Influence of faculty support initiatives and efficacy of job responsibilities. A survey data ”

Thank you.

Yours faithfully,


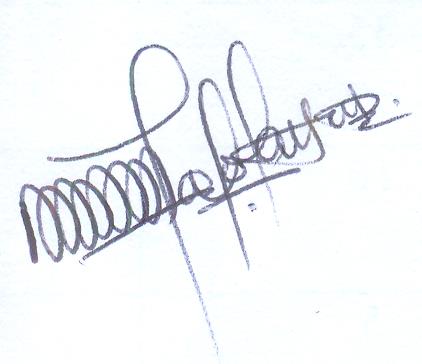


FALOLA H.O (PhD)

**Corresponding Author**
