# Supplementary material for: Data on perception of faculty members on the influence of faculty support initiatives on the efficacy of job responsibilities
Source: Data Brief. 2018 Jun 22;19:1594–9. doi: 10.1016/j.dib.2018.06.065 (PMC6141862; doi:10.1016/j.dib.2018.06.065)
Supplement: Supplementary file 2 — Supplementary material [file mmc2.docx]

**RESEARCH QUESTIONNAIRE**

Department of Business Management

Covenant University

Ota. Ogun State

November 14, 2017.

Dear Respondent,

I am conducting a research on “influence of Faculty Support Initiatives and Efficacy of Job Responsibilities”. The research is primarily for academic purpose and all responses shall be treated with utmost confidentiality. I therefore solicit your co-operation by your honest response to these questions.

Thank you.

Yours faithfully,

Falola Hezekiah Olubusayo

**SECTION A: RESPONDENT BIO DATA**

**Instruction:** Please mark (√**)** as appropriate in the boxes provided as required.

1. **SEX**: Male [ ]   Female [ ]
2. **AGE**: 18 – 30 [ ] 31 – 40 [ ]    41 – 50 [ ] 51 and above [ ]
3. **MARITAL STATUS**: Single [ ] Married [ ] Others [ ]
4. **CURRENT RANK/ LEVEL IN THE UNIVERSITY**:

Prof. [ ] Associate Prof. [ ] Snr. Lecturer [ ] Lecturer I [ ]

Lecturer II [ ] Asst. Lecturer [ ] Graduate Asst. [ ]

1. **CURRENT ADMINISTRATIVE POSITION**: (e.g Registrar, Dean, HOD, etc.)______________
2. **WORK EXPERIENCE**:

1-10years [ ] 11-20years [ ] 21years and above [ ]

1. **Please state your current university: ____________________________________**

**SECTION B**

**Please tick the appropriate option where *SA- Strongly Agree, A- Agree, U- Undecided, D- Disagree, SD- Strongly Disagree***

**Faculty Support Initiatives**

| **S/N** | **VIEW** | **SA** | **A** | **U** | **D** | **SD** |
| --- | --- | --- | --- | --- | --- | --- |
| 1 | My university encourage faculty members to attend conferences by given them full sponsorship |  |  |  |  |  |
| 2 | Conference attendance is one of the criteria for promotion |  |  |  |  |  |
| 3 | I attend conference every year |  |  |  |  |  |
| 4 | My university gives research grants for clusters |  |  |  |  |  |
| 5 | My university is always ready to sponsor research endeavours |  |  |  |  |  |
| 6 | My cluster have enjoyed grant from my university |  |  |  |  |  |
| 7 | My university pays for article processing charges |  |  |  |  |  |
| 8 | I have enjoyed publication support from my university |  |  |  |  |  |
| 9 | The level of publication support given by my university is very encouraging |  |  |  |  |  |

Efficacy of Job Responsibilities

| **S/N** | | **VIEW** | **SA** | **A** | **U** | **D** | **SD** |
| --- | --- | --- | --- | --- | --- | --- | --- |
| 10 | | My university gives opportunity for postdoc scheme |  |  |  |  |  |
| 11 | | My University allows faculty to go for research leave with pay |  |  |  |  |  |
| 12 | | I have been privilege to go for research leave |  |  |  |  |  |
| 13 | | I am encouraged to provide valuable advices to my students |  |  |  |  |  |
| 14 | | I am serving in one committee or the other |  |  |  |  |  |
| 15 | | The level of administrative engagement is very encouraging |  |  |  |  |  |
| 16 | | My teaching schedule is adequate |  |  |  |  |  |
| 17 | | I enjoy teaching because of the availability of modern teaching facilities |  |  |  |  |  |
| 18 | | I am motivated to teach |  |  |  |  |  |
| 19 | I publish minimum of 3 articles in Scopus indexed journals per year | |  |  |  |  |  |
| 20 | I publish more in international journals | |  |  |  |  |  |
| 21 | My google scholar and Scopus citations are very encouraging | |  |  |  |  |  |
